# The Discovery, Molecular Cloning, and Characterization of Dextransucrase *Lm*DexA and Its Active Truncated Mutant from *Leuconostoc mesenteroides* NN710

**DOI:** 10.3390/molecules29133242

**Published:** 2024-07-08

**Authors:** Xiaoqiong Zuo, Lixia Pan, Wenchao Zhang, Jing Zhu, Yan Qin, Xiuying Xu, Qingyan Wang

**Affiliations:** 1 National Key Laboratory of Non-Food Biomass Energy Technology, Guangxi Academy of Sciences, Nanning 530007, China; zxq20150927@163.com (X.Z.); panlixia@gxas.cn (L.P.); seven_0807@163.com (W.Z.); flyswallow2001@163.com (Y.Q.); xuxiuyingchina@163.com (X.X.); 2 College of Food and Quality Engineering, Nanning University, Nanning 530200, China; zj39330@163.com

**Keywords:** dextransucrase, N-terminal truncation, dextran, *Leuconostoc mesenteroides*

## Abstract

Dextransucrases play a crucial role in the production of dextran from economical sucrose; therefore, there is a pressing demand to explore novel dextransucrases with better performance. This study characterized a dextransucrase enzyme, *Lm*DexA, which was identified from the *Leuconostoc mesenteroides* NN710. This bacterium was isolated from the soil of growing dragon fruit in Guangxi province, China. We successfully constructed six different N-terminal truncated variants through sequential analysis. Additionally, a truncated variant, ΔN190*Lm*DexA, was constructed by removing the 190 amino acids fragment from the N-terminal. This truncated variant was then successfully expressed heterologously in *Escherichia coli* and purified. The purified ΔN190*Lm*DexA demonstrated optimal hydrolysis activity at a pH of 5.6 and a temperature of 30 °C. Its maximum specific activity was measured to be 126.13 U/mg, with a *K*_m_ of 13.7 mM. Results demonstrated a significant improvement in the heterologous expression level and total enzyme activity of ΔN190*Lm*DexA. ΔN190*Lm*DexA exhibited both hydrolytic and transsaccharolytic enzymatic activities. When sucrose was used as the substrate, it primarily produced high-molecular-weight dextran (>400 kDa). However, upon the addition of maltose as a receptor, it resulted in the production of a significant amount of oligosaccharides. Our results can provide valuable information for enhancing the characteristics of recombinant dextransucrase and potentially converting sucrose into high-value-added dextran and oligosaccharides.

## 1. Introduction

Dextransucrase is an essential enzyme in the field of dextran research. It belongs to the glycoside hydrolase enzymes (GH70) [1,2]. There are four main types of GH70 glucansucrases: alternansucrase [3], dextransucrase [4], mutansucrase [5], and reuteransucrase [6]. These enzymes produce different types of glucans with varying glycosidic linkages. Dextran, also known as glucan, is a complex polysaccharide consisting of D-glucose units connected by α-1→2, α-1→3, α-1→4, and α-1→6 glycosidic linkages [7,8]. Dextran has gained popularity due to its unique physicochemical properties, molecular size, and structure. It finds widespread applications in various industries such as pharmaceuticals, diagnostics, cosmetics, nanotechnologies, mineral extractions, and foods [3,9,10,11,12]. In fact, dextran was the first α-glucan to be industrially used as a blood plasma substitute in the 1940s [13]. Moreover, dextrans can be directly added to fermented food products to enhance texture and provide functional properties. They are also utilized as prebiotic ingredients, promoting the growth of beneficial gut bacteria [14].

Van Tieghem et al. isolated the first dextransucrase from *Leuconostoc mesenteroides* in 1878, and then named the extracellular enzyme that produces dextran from sucrose as “dextransucrase” [15]. Dextransucrase is a large multidomain glucansucrase enzyme, which is produced by various strains of *L. mesenteroides* [16], *Lactobacillus*, *Streptococcus, Anomococcus*, *Neisseri*, *Alternating monomonas*, *Pseudomonas*, *Bifidobacterium*, etc. [17,18]. *L. mesenteroides* NRRL B-1355 dextransucrase has been the focus of much attention and its resulting dextran is produced commercially [19]. The dextransucrase synthase molecule consists of 50 to 1600 amino acids, and includes a signal peptide and variable regions in the N-terminus. The N-terminal catalytic region is the key component, as it covalently bonds and breaks down sucrose into D-glucosyl-enzyme. The C-terminal dextran binding region binds to the glucose group, aiding in the elongation of the dextran chain [20].

In April 2023, 952 sequences were recorded in the GH70 family of CAZy (Carbohydrate Active enzymes) database compared to only 790 in April 2021 [21,22]. However, as of now, only 61 enzyme have been biochemically characterized. This number is quite small, and it is highly likely that many more novel and unique enzymes will be discovered in the near future. To date, the database records 43 enzymes classified as dextransucrases, α-1,6-glucosyltransferases, or α-1,6/α-1,3-glucosyltransferases that are still overrepresented in the GH70 family [23]. Native dextransucrases typically have low stabilities, and previous studies have explored various methods to enhance their stability. These methods include removing the sensitive domain [24], introducing molecular chaperones [25], truncation, and cyclization [26].

Finding new sources of enzyme production and improving the production of heterologously expressed dextransucrase for dextran synthesis has generated significant scientific and economic interest. In this study, a sucrose-degrading strain, which synthesizes dextran, was obtained from local soil in Guangxi province. China. According to the results of its morphology and 16S rDNA sequences, the strain was identified as *L. mesenteroides* NN710. To enhance the yield of heterologous recombinant production and maximize the utilization of sucrose for the production of high-value dextran, it is crucial to acquire more efficient dextransucrases with superior enzymatic properties. In this study, we constructed *Lm*DexA and multiple N-terminal truncation mutants of *Lm*DexA from *L. mesenteroides* NN710. Specifically, we carried out cloning and expression of *Lm*DexA and its variants, including ΔN20*Lm*DexA, ΔN39*Lm*DexA, ΔN99*Lm*DexA, ΔN148*Lm*DexA, ΔN190*Lm*DexA, and ΔN280*Lm*DexA. The expression was achieved using an *E*. *coli* Rosetta (DE3) expression system. The ΔN190*Lm*DexA was successfully purified and subjected to biochemical characterization. The results obtained demonstrated a significant improvement in the level of heterologous expression and total enzyme activity of ΔN190*Lm*DexA. Additionally, when the products of ΔN190*Lm*DexA were analyzed using HPLC, it was evident that ΔN190*Lm*DexA displayed both hydrolytic and transsaccharolytic activities.

## 2. Materials and Methods

### 2.1. Medium and Plasmid

The *E. coli* strains JM110, Rosetta (DE3), and the plasmid pET-30a (+) were stored in our laboratory. The plasmid pET-30a (+) and *E. coli* Rosetta (DE3) were used for dextransucrase expression. The recombinant strain was cultivated aerobically at 37 °C in Luria–Bertani (LB) broth that was supplemented with 50 kanamycin μg/mL. The composition of the LB was as follows: 10 g/L of tryptone, 5 g/L of yeast extract, and 10 g/L of NaCl, with a pH 7.0. LB liquid medium was added with 20 g/L agar powder to obtain solid LB medium.

### 2.2. Isolation and Identification of Strain

The strain was isolated from soil samples collected in Guangxi province, China (107°69′19″ E 23°17′33″ N). To culture the samples, they were diluted and spread on LB broth supplemented with 68% sucrose and 2.0% agar. The plates were then incubated at 30 °C for 2 days. The strain that exhibited a high level of viscous slimy growth on sucrose agar plates, along with the highest crude polysaccharide production as measured using the phenol-sulfuric acid method [27,28], was selected as the target strain.

The strain was cultured in MRS broth in an incubator of 200 rpm for 2 days at 30 °C. The composition of the MRS broth was as follows: 10 g/L tryptone, 4 g/L yeast extract, 8 g/L meat extract, 20 g/L glucose, 5 g/L NaAc, 1 g/L tween 80, 2 g/L KH_2_PHO_4_, 2 g/L C_6_H_17_N_3_O_7_, 0.2 g/L MgSO_4_·7H_2_0, and 0.05 g/L MgSO_4_·4H_2_O, with a pH of 6.7. The total genomic DNA of the screening strain was extracted using bacteria DNA Isolation Mini kit (Vazyme, Nanjing, China), and identified by 16S rRNA gene sequencing analysis using universal primers [29]. The gene sequences were compared by using BLAST searches of the GenBank database to identify closest relativities. The phylogenetic tree was generated using MEGA 5.0 software [30].

### 2.3. Cloning of Dextransucrase and Its Truncated Variants Genes

The primers for the dextransucrase and its truncated variants genes were designed according to the sequence of the *L. mesenteroides* gene (AY017384.11) available in the NCBI database. Using the genome of strain NN710 as a template, a 4700 bp fragment containing the entire dextransucrase gene was amplified by polymerase chain reaction (PCR) using designed primers (*Lm*DexAP1: 5′-CAGTCATGAACATTTACAGAAAAAGTAATGCGG-3′, *P*agI restriction site, *Lm*DexAP2: 5′-GTGGAGCTCCCGAAAAAGAAATGAATAAA-3′, *S*acI restriction site). PCR was carried out as follows: 1 cycle at 95 °C for 5 min, followed by 32 cycles at 95 °C for 30 s, 55 °C for 2 min, and 72 °C for 10 min using DNA Polymerase (2× Phanta Max Master Mix, Nanjing, China).

The nucleotide sequence of the dextransucrase gene has been uploaded in GenBank under the accession number OP778186.2. N-terminal truncated mutants were amplified using the primer sets listed in Table 1. The purified dextransucrase fragment was ligated into pET-30a (+) that had been previously digested with *P*agI and *S*acI using T4 DNA ligase (Vazyme, Nanjing, China), resulting in the creation of the recombinant plasmid pET-30a (+)-*Lm*DexA. Other expression plasmids pET-30a (+)-ΔN20*Lm*DexA, pET-30a (+)-ΔN39*Lm*DexA, pET-30a (+)-ΔN99*Lm*DexA, pET-30a (+)-ΔN148*Lm*DexA, pET-30a (+)-ΔN190*Lm*DexA, and pET-30a (+)-ΔN280*Lm*DexA were constructed by the same strategy. The recombinant plasmids were transformed into the cloning host *E.coli* JM110. Subsequently, the plasmid pET-30a (+) and the correct recombinant plasmids were further transformed into the expression host *E.coli* Rosetta (DE3).

### 2.4. Protein Expression and Purification of the Recombinant Dextransucrases

The recombinant plasmids pET-30a (+)-*Lm*DexA, pET-30a (+)-ΔN20*Lm*DexA, pET-30a (+)-ΔN39*Lm*DexA, pET-30a (+)-ΔN99*Lm*DexA, pET-30a (+)-ΔN148*Lm*DexA, pET-30a (+)-ΔN190*LmdexA,* and pET-30a (+)-ΔN280*Lm*DexA were, respectively, transformed into the expression hosts *E. coli* Rosetta (DE3). The transformed expression hosts were then transferred into 2 mL fresh LB medium with 50 μg/mL kanamycin. The culture was incubated at 37 °C and 220 rpm for about 3 h, until the optical density at OD_600_ reached approximately 0.5–0.8. The isopropyl β-D-1-thiogalactopyranoside (IPTG) was added to a final concentration of 0.8 mM. The cells were subsequently cultured for 4 h at 20 °C [31], and collected through centrifugation at 6000 rpm for 10 min at 4 °C. Sequentially, the mixture was disrupted through ultrasonication (KS-1000ZDN, Kunshan, China) on ice. The supernatant was obtained by centrifugation at 4 °C for 1 h (12,000 rpm). The crude enzyme was loaded onto a column containing Ni-NTA, which was purchased from CowinBio (Taizhou, China). Contaminating proteins were removed by washing with a buffer solution of 20 mM NaAc, 0.5M NaCl, 50 mM imidazole, and pH 5.6. The dextransucrase protein was subsequently eluted using an elution buffer containing 20 mM NaAc, 0.5M NaCl, 200 mM imidazole, and pH 5.6. The eluted protein was collected by the self-flow of an imidazole solution under gravity, followed by concentration and desalting using a 30 kDa centrifuge ultrafiltration tube (Millipore, Danvers, MA, USA) with a 20 mM sodium acetate buffer at pH 5.6. SDS-PAGE analysis confirmed that ΔN190*Lm*DexA pure is pure to homogeneity.

### 2.5. Enzyme Activity Assays

Depending on the receptors, dextransucrase catalyzes two kinds of reactions: hydrolysis and glycosyl transfer. The dextransucrase activity of *Lm*DexA was detected by measuring the release of reducing sugars in the presence of sucrose. Fructose is a reducing sugar, and its concentration was determined using the DNS (3,5-dinitrosalicylic acid) method [32,33,34]. One unit of enzyme activity was specified as the amount of enzyme that generates 1 µmol of fructose per minute at a temperature of 30 °C in a sodium acetate buffer with a pH 5.6, with a concentration of 20 mM [35]. The reaction system consisted of 990 µL of pH 5.8 sodium acetate buffer (20 mM) containing 68 g/L sucrose, and 10 µL (0.6 mg/mL) of purified ΔN190*Lm*DexA. The total volume of the reaction system was 1 mL. The reaction period lasted for 10 min at a temperature of 30 °C. After the reaction, the sample was heat-treated at a temperature of 90 °C for 5 min to inactivate any remaining enzymes. Following the inactivation step, the enzyme activity was determined by measuring the absorbance at OD_540_ [35,36].

### 2.6. Biochemical Characterization of Recombinant Dextransucrase

The optimal temperature and pH should be determined by conducting measurements at various temperatures, ranging from 20 to 40 °C, and pH levels, ranging from 3.0 to 5.8. Under optimal reaction conditions, we determined the temperature stability of ΔN190*Lm*DexA at different temperatures (30–40 °C) for 1 h. Additionally, we examined the pH stability of ΔN190*Lm*DexA at different pH values (3.6–8.0) for 24 h. Specifically, we evaluated the stability of ΔN190*Lm*DexA in NaAc-HAc buffer with a pH range of 3.0–5.8 and in NaH_2_PO_4_-Na_2_HPO_4_ buffer with a pH range of 5.8–8.0. The enzyme activity that exhibited the greatest value was considered as 100%, whereas the activities at other conditions were expressed as a ratio to this maximum activity.

The influences of metal ions (Ca^2+^, Co^2+^, Zn^2+^, Cu^2+^, K^+^, Na^+^, Mn^2+^, Mg^2+^) on the activities of ΔN190*Lm*DexA were measured under optimum temperature and pH conditions. The dextransucrase activity in the presence of 10 mM metal ions was compared to the control, which had no added metal ions.

The enzyme activities were determined at various sucrose concentrations ranging from 3.0 mM to 800 mM, at a pH of 5.6 and a temperature of 30 °C, over a duration of 30 min. The optimal concentration of substrate was determined based on the highest enzyme activity observed. The Michaelis–Menten kinetic constant was determined using sucrose as a substrate to assess the catalytic efficiency of the ΔN190*Lm*DexA. The Michaelis constant, *K_m_*, and maximum velocity, *V_max_*, were determined by measuring the reaction rate at various sucrose concentrations ranging from 3 mM to 300 mM. All experiments were carried out under identical conditions. These parameters were calculated using Michaelis–Menten kinetic equations, unless otherwise stated. The calculations were performed using the program Origin 8.0.

### 2.7. Product Analysis

Dextransucrase catalyzes have two kinds of reactions, including hydrolysis and glycosyl transfer. ΔN190*Lm*DexA catalyzed the transfer of D-glucosyl units from sucrose to acceptor molecules, and maltose were used as acceptors. The reaction products formed by the hydrolysis of sucrose using ΔN190*Lm*DexA were analyzed using silica gel thin-layer chromatography (TLC) on a GF254 plate (Qingdao, China). The reaction mixture was carried out following the established method with a few minor modifications. The reaction containing 990 µL of 6.8% sucrose and 10 µL of ΔN190*Lm*DexA dissolved in a 20 mM NaAc–HAc buffer (pH 5.6) was incubated at 30 °C for 10, 20, and 30 min. The reaction mixture was heated for 5 min in boiling water, followed by centrifugation at 12,000 rpm for 30 min at 25 °C. The resulting supernatant was analyzed by TLC using a solvent mixture consisting of n-butanol, acetic acid, and water in a ratio of 2:1:1. The visualization of the products can be achieved through the use of a spraying method. This method involved evenly spraying a reagent called diphenylamine-aniline-phosphate onto the plate. The reagent was a mixture consisting of 1 mL of aniline, 1 g of diphenylamine, 5 mL of concentrated phosphoric acid, and 50 mL of acetone. After the spraying process, the plate was then heated at a temperature of 50 °C for 30 min. Biotransformation of dextransucrase resulted in the conversion of sucrose to dextran and fructose within 4 h, as determined by HPLC analysis. HPLC conditions included a TSKgel column, ultrapure water used as the mobile phase, a flow rate of 0.6 mL/min, an injection volume of 10 μL, a detector temperature maintained at 40 °C, and column temperature set at 60 °C. Fructose was quantified using the DNS method.

## 3. Results and Discussion

### 3.1. Screening of the Strains Manifesting Dextran Synthesis Ability

**Figure 1:**
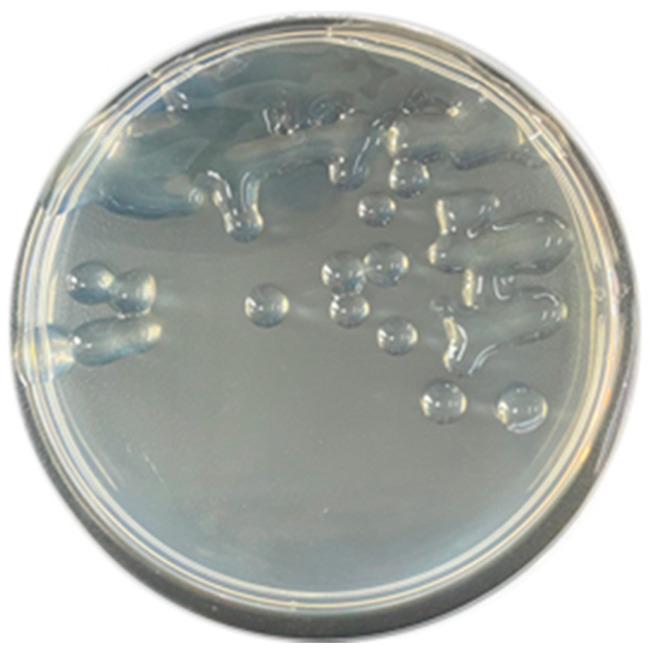
Shiny growth of NN710 on sucrose-containing medium plate.

Colonial growth that exhibited a highly viscous slimy appearance on a sucrose agar plate was selected using an enrichment culture method. Following the initial screening and subsequent rescreening processes, ten colonies were identified from various soil samples. These colonies were found to produce a viscous extracellular polysaccharide when cultured on a medium containing 68% sucrose. The crude polysaccharide production was measured using the phenol-sulfuric acid method, and the extent of mucilage production on the agar plate was evaluated. The strain with the number NN710, which exhibited the desired characteristics, was selected as the target strain for further identification and analysis. The strain NN710 streaked repeatedly on the agar plates (Figure 1).

### 3.2. The Identification of the Strain NN710

**Figure 2:**
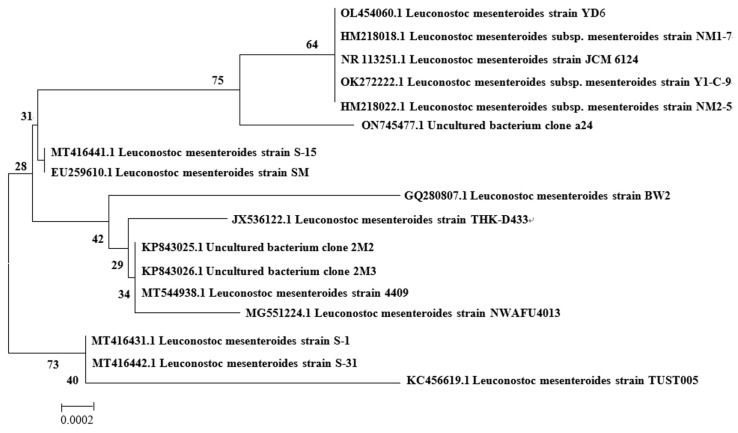
Phylogenetic tree of strain *Leuconostoc mesenteroides* NN710.

The strain NN710 16S rDNA gene sequences were amplified using primers (27F: 5′-AGAGTTTGATCMTGGCTCAG-3′, 1492R: 5′-GGTTACCTTGTTACGACTT-3′) and then sequenced by Sheng Gong (Shanghai, China). In order to identify the closest known relatives, the gene sequences were compared through BLAST searches in the GenBank database. The phylogenetic tree was generated using MEGA 5.0 software [30], as shown in Figure 2. It can be seen that strain NN710 has the highest homology of 99% with *L. mesenteroides* strain S-31 (MT416442.1). Combined with the results of its morphological observation, the strain was determined to be *L. mesenteroides* NN710.

### 3.3. Sequence Analysis of LmDexA

**Figure 3:**
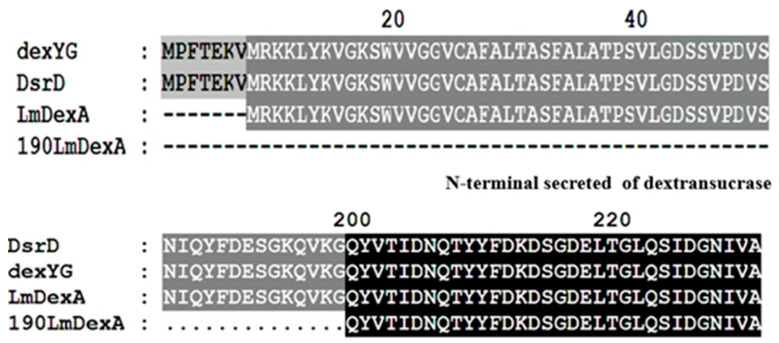
Alignment of *Lm*DexA amino acid sequence with other dextransucrases from different *L. mesenteroides.* DsrD from *L. mesenteroides Lcc*4 (GenBank No: AY017384), dexYG from *L. mesenteroides 0326* (GenBank No: DQ345760), and *Lm*DexA from the *L*. *mesenteroides* NN710 (GenBank No: OP778186.2). The truncated parts of *Lm*DexA are shown against a grey background.

The amino acid sequence of *Lm*DexA showed similarities to conserved regions of dextransucrases when compared to other proteins using a BLAST similarity search in the GenBank database (http://www.ncbi.nlm.nih.gov.cn/BLAST, accessed on 12 December 2023). The highest level of homology was found with DsrD (GenBank No: AY017384) from *L. mesenteroides Lcc*4 (99.8% identity). *Lm*DexA also exhibits significant similarities to other dextransucrases, including dexYG. Additionally, the conserved residues identified in the N-terminal domain of these dextransucrases were also conserved in *Lm*DexA (Figure 3). The signal peptidase cleavage site at residues between position 35 and 36 (MRKKLYKVGKSWVVGGVCAFALTASFALATPSVLG-DSSV) was predicted using SignalP 5.0 server (www.cbs.dtu.dk/services/SignalP, accessed on 12 December 2023). The amino acid sequence of *Lm*DexA is highly similar to DsrD and dexYG.

### 3.4. N-Terminal Truncation Construction Strategy

**Figure 4:**
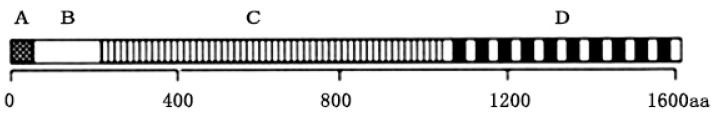
The domain architecture analysis of dextransucrases *Lm*DexA. A, signal peptide; B, variable region; C, N-terminal catalytic domain; D, C-terminal glucan binding domain.

The alignment of the amino acid sequence analysis showed that *Lm*DexA contains the catalytic signature motif of GH70. The protein molecule consists of four regions, as shown in Figure 4. Regions A and B correspond to the signal peptide and variable region, respectively. Regions C and D represent the N-terminal catalytic region involved in the binding and separation of sucrose, and the C-terminal dextran binding region responsible for dextran chain binding. Region C, known as the core region, is where the enzyme binds to sucrose through covalent bonding and decomposes it into D-glucosyl-enzyme. This enzymatic formation is crucial for the process [20,37,38,39]. The nonconserved region located immediately downstream of the signal peptide does not seem to have a significant impact on the enzyme’s functioning. Therefore, removing it may not have any detrimental impact on the activity of the enzyme [23]. To enhance the expression level of *Lm*DexA, we sought to investigate the impact of deleting the N-terminal flexible region and signal peptide in *Lm*DexA on protein expression and purification. Moreover, DSR-A, which is isolated from *L. mesenteroides* NRRL B-1299, is an active enzyme that does not possess this variable region [35]. This variance in domain architecture ultimately leads to a wide diversity in the size of these enzymes, ranging between 120 and 200 kDa, with a few exceptions. For example, dextransucrases from *L. mesenteroides* NRRL B-1299 contain four enzymes with molecular masses ranging from 195 to 283 kDa [20,40].

**Figure 5:**
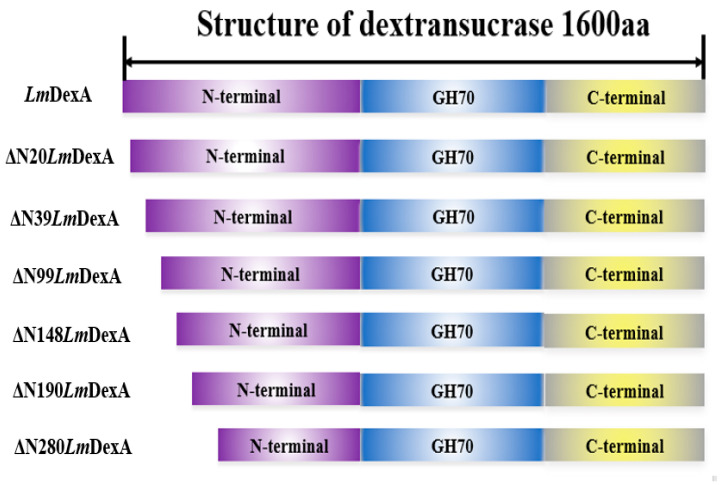
Schematic representation of N-terminal truncation variants.

In general, introducing mutations in the flexible region of an enzyme can enhance its activity. Moreover, truncating the enzyme may modify the specificity of the products generated [23,38]. In this study, our primary objective was to enhance the heterologous expression level and enzyme activity of recombinant dextransucrase through truncation of its N-terminus. Specifically, we created six mutant strains by deleting the N-terminal fragment, which ranged from 60 to 840 base pairs. According to the amino acid number, the recombinant proteins were designated as ΔN20*Lm*DexA, ΔN39*Lm*DexA, ΔN99*Lm*DexA, ΔN148*Lm*DexA, ΔN190*Lm*DexA, and ΔN280*Lm*DexA, shown in Figure 5.

### 3.5. Expression and Purification of N-Terminal Truncation Mutants

**Figure 6:**
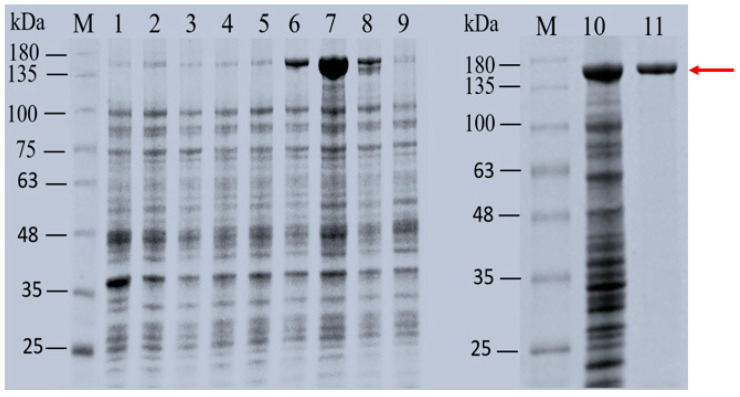
SDS-PAGE analysis of *Lm*DexA and six truncated variants. **M**, protein molecular weight markers; **1**, soluble intracellular protein of induced of *E.coli* Rosetta (DE3)/pET-30a(+); **2**, soluble intracellular protein of induced of *E. coli* Rosetta (DE3)/pET-30a(+)-*Lm*DexA; **3**, soluble intracellular protein of induced of *E. coli* Rosetta (DE3)/pET-30a(+)ΔN20*Lm*DexA; **4**, soluble intracellular protein of induced of *E. coli* Rosetta(DE3)/pET-30a(+)-ΔN39*Lm*DexA; **5**, soluble intracellular protein of induced of *E. coli* Rosetta (DE3)/pET-30a(+)-ΔN99*Lm*DexA; **6**, soluble intracellular protein of induced of *E. coli* Rosetta (DE3)/pET-30a(+)-ΔN148*Lm*DexA; **7** and **10**, soluble intracellular protein of induced of *E. coli* Rosetta (DE3)/pET-30a(+)-ΔN190*Lm*DexA; **8**, soluble intracellular protein of induced of *E. coli* Rosetta (DE3)/pET-30a(+)-ΔN280*Lm*DexA; **9**, soluble intracellular protein of induced of *E. coli* Rosetta (DE3)/pET-30a(+)-ΔN190*Lm*DexA (without IPTG); **11**, purified ΔN190*Lm*DexA, marked with the red arrow.

The gene sequences of ΔN20*Lm*DexA, ΔN39*Lm*DexA, ΔN99*Lm*DexA, ΔN148*Lm*DexA, ΔN190*Lm*DexA, and ΔN280*Lm*DexA were obtained from NN710 and subsequently cloned into *E. coli* Rosetta (DE3) for successful protein expression. The expression levels of *Lm*DexA, ΔN20*Lm*DexA, ΔN39*Lm*DexA, ΔN99*Lm*DexA, ΔN148*Lm*DexA, ΔN190*Lm*DexA, and ΔN280*Lm*DexA were analyzed using SDS-PAGE (Figure 6). The ΔN190*Lm*DexA exhibited the highest level of expression and was encoded by a recombinant gene consisting of 1372 amino acid residues, with a theoretical molecular weight of 150 kDa. By measuring the enzymatic activities of *Lm*DexA and its N-terminal truncation six mutants at 30 °C, we observed that the mutant ΔN190*Lm*DexA exhibited the highest enzymatic activity. In particular, the ΔN190*Lm*DexA mutant showed a volumetric activity of 107 U mL^−1^ in the crude enzyme extracts after a 4 h induction of recombinant expression. The crude activity of ΔN190*Lm*DexA (107.7 U mL^−1^) is higher compared to the reported recombinant dextransucrases from other strains of *L. mesenteroides* (Table 2).

### 3.6. Effects of pH and Temperature on Activity and Stability of ΔN190LmDexA

The ΔN190*Lm*DexA gene was successfully cloned into the plasmid pET-30a (+) and demonstrated efficient expression in *E. coli*. It is stable under acidic conditions but easily denatured under neutral and alkaline conditions. We used acidic conditions at pH 5.6, where the yield of ΔN190*Lm*DexA was 35.23% for further experiments. The purified product exhibited a 19.37-fold increase in yield and achieved a specific activity of 126.13 U/mg (Table 3). Therefore, we successfully purified a significant quantity of the ΔN190*Lm*DexA recombinant protein and advanced to the subsequent phase of the study.

**Figure 7:**
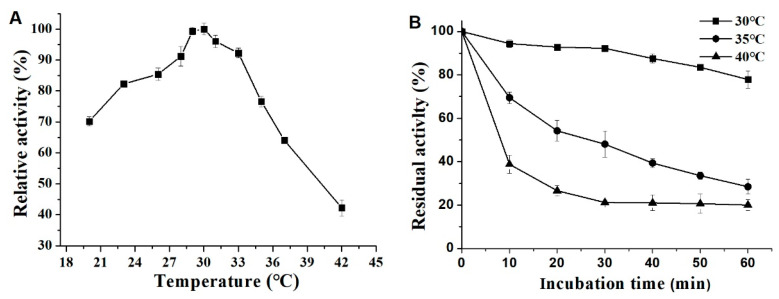

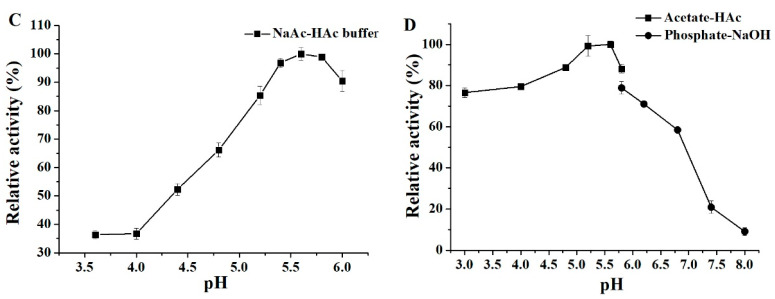
Effects of temperature and pH on the activity and stability of ΔN190*Lm*DexA. (**A**) The optimum temperature of ΔN190*Lm*DexA. (**B**) Effects of temperature on enzyme stability of ΔN190*Lm*DexA. (**C**) The optimum pH of ΔN190*Lm*DexA. (**D**) Effects of pH on enzyme stability of ΔN190*Lm*DexA.

We explored the effects of pH and temperature on the enzyme activity and stability of ΔN190*Lm*DexA under different pH and temperatures. The results indicated that the optimum temperature for ΔN190*Lm*DexA activity was approximately 30 °C when sucrose was used as the substrate, as shown in Figure 7A. The thermostability of ΔN190*Lm*DexA was assessed by preincubating it at various temperatures in the absence of substrate for 60 min. It was observed that the relative activity of ΔN190*Lm*DexA decreased below 35 °C, with a relative activity of only 40% after 60 min. However, at 30 °C, over 80% of the relative activity was maintained for the entire 60 min period (Figure 7B). This suggests that ΔN190*Lm*DexA exhibits good temperature stability, particularly at room temperature. The effect of pH on the enzyme activity of ΔN190*Lm*DexA was observed, showing that it reached peak activity at pH 5–6, with an optimal pH of 5.6 (Figure 7C). This indicates that ΔN190*Lm*DexA is an acidic enzyme. The stability of ΔN190*Lm*DexA was also investigated across a pH range of 3.6–8.0 (Figure 7D). The results showed that ΔN190*Lm*DexA was highly stable at pH 5.0–6.0, with over 80% of its activity retained after 24 h of incubation at 4 °C and pH 5.6.

NiΔN190*Lm*DexA represents purification of ΔN190LmDexA following nickel affinity chromatography.

### 3.7. Effect of Metallic Cations on ΔN190LmDexA Activity

**Figure 8:**
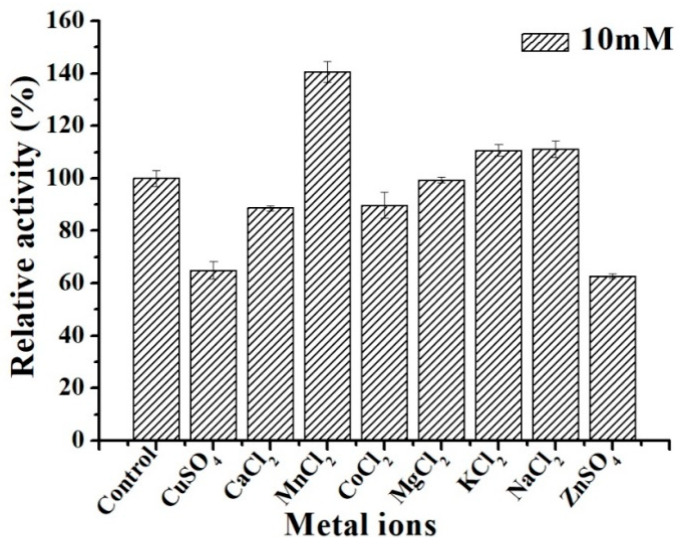
Effects of various metallic cations on activity of ΔN190*Lm*DexA.

The effect of various metallic cations on the activity of purified ΔN190*Lm*DexA is shown in Figure 8. It can be observed that the activity of ΔN190*Lm*DexA was hindered when exposed to Cu^2+^ and Zn^2+^ concentrations of 10 mM, resulting in a relative activity of less than 70%. Conversely, the addition of Mn^2+^ in the form of MnCl_2_ led to an overall increase of 40% in the activity of ΔN190*Lm*DexA. The activity of ΔN190*Lm*DexA was not significantly influenced by other cations such as Ca^2+^, K^+^, and Na^+^. However, when Ca^2+^ is present within a specific concentration range, it exhibits a preference for binding to the activation site on the enzyme. This leads to a stronger activation effect as compared to inhibition [47]. Our experimental results show that the concentration of Ca^2+^ is 10 mM at the inhibited concentration.

### 3.8. Effect of Substrate Concentration and Kinetic Parameters of ΔN190LmDexA

**Figure 9:**
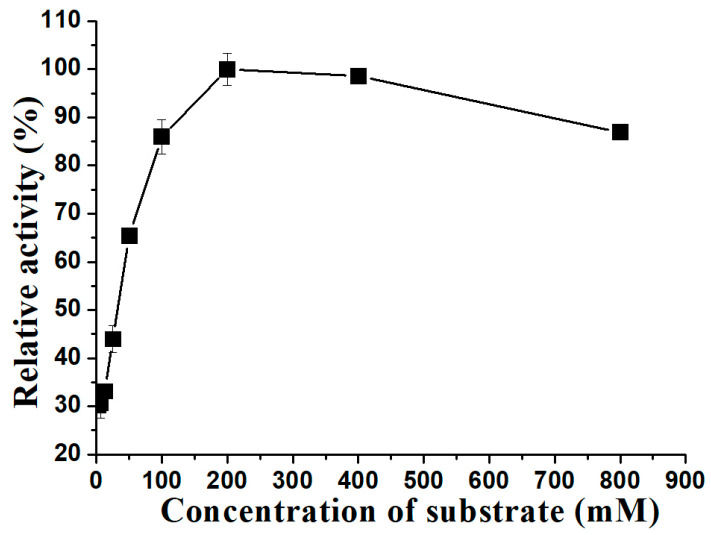
Effects of sucrose substrate on stability of ΔN190*Lm*DexA. Hydrolytic activity of ΔN190*Lm*DexA at different sucrose concentration at 30 °C.

Enzyme activities were determined on sucrose concentrations ranging from 3.0 to 800 mM at pH 5.6 and 30 °C for a duration of 30 min. The results showed that the optimal concentration of substrate was found to be 200 mM. However, as the sucrose concentration increased, the enzyme activity gradually decreased (Figure 9). The kinetic constants (Michaelis–Menten kinetics constant, *K*_m_, *V*_max_, *k*_cat_, and *k*_cat_/*K*_m_) were evaluated by measuring the reaction rates at different concentrations of sucrose as a substrate. The purified ΔN190*Lm*DexA exhibited a *K_m_* value of 13.7 mM, a *V_max_* value of 85.0 µmol/(mg∙min), a *k*_cat_ value of 141.6 s^−1^, and a *k*_cat_/*K*_m_ value of 10.3 s^−1^mM^−1^. The *K_m_* value of ΔN190*Lm*DexA was lower than that of dexYG, indicating that ΔN190*Lm*DexA has a higher affinity for the substrate. Additionally, the optimal concentration of substrate for ΔN190*Lm*DexA was similar to that of dexYG, suggesting that ΔN190*Lm*DexA is more efficient in catalyzing the substrate compared to dexYG [31,37].

### 3.9. Properties of the ΔN190LmDexA

**Figure 10:**
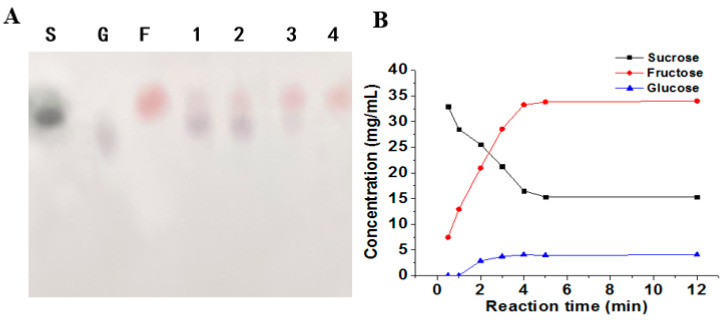
Analysis of the hydrolysis and transglycosylation products of ΔN190*Lm*DexA. (**A**) TLC analysis was performed to investigate the degradation products of ΔN190*Lm*DexA. The samples were treated with sucrose for different time durations (1–4 h). TLC analysis was performed to study the degradation products of ΔN190*Lm*DexA. In the TLC plate, different spots were used to represent different sugars. Specifically, S, sucrose; G, glucose; F, fructose. (**B**) Sucrose, glucose, and fructose were measured by HPLC analysis using a standard curve for quantitative analysis.

The reaction mixture consisted of 10 U of enzyme solution and 990 µL of 200 mM sucrose dissolved in 20 mM NaAc–HAc buffer (pH 5.6). The mixture was incubated at 30 °C for different durations. We analyzed the reaction products using TLC assay and found that fructose was observed after 1 h of incubation. The amount of fructose gradually increased with longer incubation times, as shown in Figure 10A. However, apart from fructose, we did not detect any other products after 4 h of incubation. We hypothesized that the concentration of glucose in the reaction system was below the minimum detection limit of the TLC method. This is because the glucose molecules were polymerized into “glucan”, also known as dextran. To confirm the presence of dextran, we detected it using HPLC.

**Figure 11:**
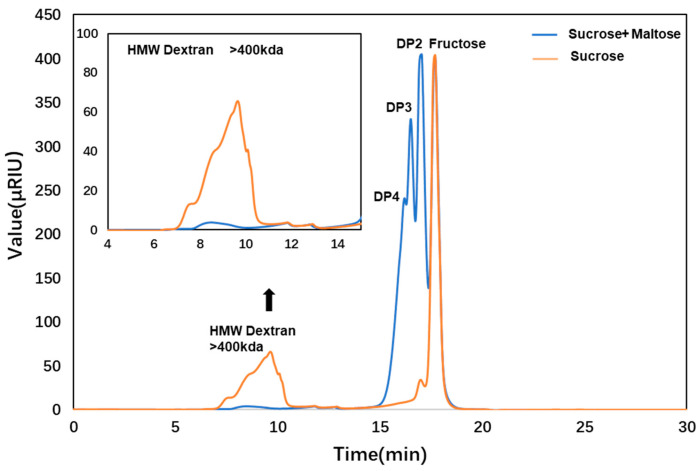
HPLC analysis of the products produced by ΔN190*Lm*DexA. The reaction conditions were as follows: 100 mM sucrose was used as the substrate after 24 h (line orange), 100 mM sucrose was used as substrate and 100 mM maltose was added as receptor after 24 h (line blue). It was observed that the retention time of the products was inversely proportional to their molecular weight. In other words, products with higher molecular weight had shorter retention times.

Further analysis of the product from the biotransformation of ΔN190*Lm*DexA using HPLC revealed that the glucose levels were significantly lower than 5 g/L in the reaction (Figure 10B). During the biotransformation test of ΔN190*Lm*DexA, the sucrose substrate was converted into dextran and fructose. After reacting with 100 mM sucrose as a substrate for 24 h, GPC results show two main peaks: HMW polysaccharides produced eluted from 5 to 10 min and fructose produced at about 17 min. In addition, a small amount of oligosaccharides with a degree of polymerization (DP) of approximately 2 was generated, eluting at 16.5 min. Receptor reaction products of ΔN190*Lm*DexA were also determined. By utilizing 100 mM sucrose as a substrate and introducing a 100 mM maltose receptor reaction for 24 h, it was observed that the yield of dextran decreased, while the quantity of oligosaccharides with DP2-DP4, eluting between 16 and 17.5 min, exhibited a notable increase. Based on the results mentioned above, ΔN190*Lm*DexA exhibited both hydrolytic and transsaccharolytic activity. When sucrose was employed as a substrate, it primarily generated high-molecular-weight dextran (>400 kDa). Upon the introduction of maltose as a receptor, a considerable portion of glucans were translocated to the receptor, forming oligosaccharides, leading to a notable reduction in dextran yield without altering its size. The resulting product bore resemblance to dexYG, a known producer of high-molecular-weight dextran [37]. These findings were confirmed through HPLC analysis (Figure 11).

## 4. Conclusions

In this work, a strain manifesting potent sucrose hydrolysis activity was isolated and screened from the soil samples collected in NanNing, Guangxi, China. This strain was identified as *L. mesenteroides* NN710 based on morphological observation and 16S rDNA sequence analysis. The gene sequence encoding the GH70 family (*Lm*DexA) in the genome of the strain was cloned. To improve protein expression in *E. coli* and enhance recombinant enzyme activity, we generated six truncations of the dextransucrases *Lm*DexA. We observed an enhancement in protein expression and activity, with a value of 107.7 U mL^−1^, after truncating the first 190 amino acids from the N-terminus of *Lm*DexA. Our findings suggest that the ΔN190*Lm*DexA variant holds promise for effective dextran and oligosaccharides synthesis in the future. This variant could potentially serve as a foundation for the continued refinement and modification of dextransucrase for various applications.

## Figures and Tables

**Table 1:** The primer sequences used to amplify the genes of the N-terminal truncated mutants of dextransucrase.

N-Terminal Truncation Primers	Sequence (5′to 3′)
pET-30a (+)-ΔN 20 *Lm*DexA	TAGTCATGAGCTTTTGCATTAACCGCCTC
pET-30a (+)-ΔN 39 *Lm*DexA	CAGTCATGACAGAACACTACGGTTACCGA
pET-30a (+)-ΔN 99 *Lm*DexA	CAGTCATGACAATCTGCTGATAATAATGTG
pET-30a (+)-ΔN148*Lm*DexA	CAGTCATGATTAGCGGCAAGTACGTTGAA
pET-30a (+)-ΔN190*Lm*DexA	TAGTCATGATCAAAGGACAGTATGTCACAAT
pET-30a (+)-ΔN280*Lm*DexA	TAGTCATGATGATTGATGGTCAAATAATGAC

**Table 2:** Comparison of the properties of *Lm*DexA and ΔN190*Lm*DexA with reported dextransucrases from *L. mesenteroides*.

Strain	Dextransucrase	Protein Size (aa)	Expression Plasmid	Expression Strain	Crude Enzyme Activity	References
NRRL B-512F	dsrS	1527	pTrc99A	DH1	0.2 U/mL	[41]
	dsrS	1527	pBad/Thio-TOPO	One Shot Top 10	5.85 U/mL	[42]
	dsrS	1527	pET-23d	BL21(DE3).	0.85 U/mL	[43]
	dsrT	1015	pET-23d	BL21(DE3)	0.17 U/mL	[43]
	dsrT5	1499	pET-23d	BL21(DE3)	1.9 U/mL	[43]
NRRL B-1299	dsrB	1508	pTrc 99A	DH1	2.0 mU/mL	[44]
	dsrE	2835	pBad/Thio-TOPO	One Shot Top 10	0.58 U/mL	[20]
	DsrM	2043	pET-55-DEST	BL21(DE3)	2.28 U/mL	[2]
	DSDP	1279	pENTR/D-TOPO	BL21(DE3)	0.75 U/mL	[2]
	Dsr-MΔ1	1411	pENTR/D-TOPO	BL21(DE3)	60 U/mL	[23]
	Dsr-MΔ2	1263	pENTR/D-TOPO	BL21(DE3)	67 U/mL	[23]
0326	dex-YG	1501	pET-28a(+)	BL21(DE3)	36 U/mL	[31]
NN710	*Lm*DexA	1562	pET-30a(+)	Rosetta (DE3)	2.49 U/mL	This work
	ΔN190*Lm*DexA	1372	pET-30a(+)	Rosetta (DE3)	107.7 U/mL	This work
CGMCC 1.544	dsrX	1522	pET-28a(+)	BL21(DE3)	8.8 U/mL	[45]
NRRL B-512F	dsrS	1527	pMM1520	*B. megaterium*	65 mU/mL	[46]
				MS941 (ΔnprM)	28.6 U/mL	[46]
*Lcc*4	dsrD	1527	pNZ124	*L. lactis* MG1363	0.8 U/mL	[33]

**Table 3:** Purification summary of ΔN190LmDexA.

Crude Enzyme Solution	Volume (mL)	Protein Concentration (mg/mL)	Specific Activity (U/mg)	Total Activity (U)	Purification (Fold)	Yield % (Total Activity)
ΔN190*Lm*DexA	4.0	16.55	6.51	430.8	1	100
NiΔN190*Lm*DexA	2.0	0.6	126.13	151.78	19.37	35.23

## Data Availability

Data will be made available on request.
